# Medical Advertisements

**Published:** 1866-01

**Authors:** N. S. Davis

**Affiliations:** Chairman


### CHICAGO MEDICAL EXAMINER.

N. S. DAVIS, M.D., EDITOR.

VOL. VII. JANUARY, 1866. NO. 1.

### Original Contributions.

### ARTICLE I.

### MEDICAL ADVERTISEMENTS.

The committee to whom was referred the subject of immoral and injurious medical advertisements, respectfully ask the privilege of making the following report:—

Of all the various methods devised for obtaining money by false pretences, none are carried on so extensively, or are so prolific of evil to the community, as that of medical advertising in newspapers, handbills, &c. The extent of this systematic swindling cannot be appreciated without a somewhat extensive investigation of the subject. The simple fact, however, that nearly all the newspapers in the country have from one to four columns occupied with this kind of advertisements, paid for at ordinary rates, and the further fact that every alternate fence-post, board, rock, or door-step in the country is covered with the same kind of literature, shows that an aggregate of several millions of dollars are expended annually for this purpose. Of course, all these *millions*, with liberal additions, are received back from such members of the community as can be deceived by pretension and falsehood. The class of medical advertisements to which we allude may be divided into three varieties: First, such as set forth the efficacy and importance of particular medicines in the cure of ordinary diseases. Second, such as represent the unusual and extraordinary skill and success of Dr. A. or Dr. B. in the treatment of this or that class of ordinary chronic diseases. And, third, such as relate directly to the treatment of the diseases and consequences of licentiousness.

The nature and objects of the first variety will be readily seen by the following extract, selected from the first advertisement of a medical character that met the eye on opening a daily newspaper:—

"Helmbold's Highly Concentrated Compound Fluid Extract Buchu. A *positive* and *specific* remedy for Diseases of the Bladder, Kidneys, Gravel, and Dropsical Swellings; for Weaknesses arising from excesses, habits of dissipation, early indiscretion, or abuse; for Decline or Change of Life, attended by indisposition to exertion, loss of memory, weak nerves, horror of disease, &c., &c."

The two leading traits of all this variety of advertisements are, first, the claim that the medicine is a "*positive and specific remedy;*" and, second, the enumeration of such a list of symptoms that every invalid will find among them some one or more which corresponds with his own, and be thereby led to imagine the medicine specially designed for his relief. The positive claim thus set up is known to be false, not only by every intelligent physician, but by the advertiser himself. But the unqualified assertion of curative properties, coupled with the ingenious enumeration of such symptoms as would apply to almost any chronic malady, is admirably calculated to win the confidence of the sick, so far as to make them desirous of giving it a trial. A trial involves the purchase of a bottle or box, as the case may be, and this accomplishes all the advertiser expects. The framing and publication of such advertisements is just as deliberate a fraud as it would be for an individual to enter a store with false testimonials of wealth and character, and thereby obtain a bill of goods on credit.

The nature and style of the second variety of advertisements is well-illustrated by the following, taken from another of our morning papers:—

"Dr. D. MacRae's Medical Institute, at 149 South Clark St.,

Chicago, Ill. P.O. Box, 2156. The above Medical Institute is intended for special treatment of all chronic diseases of the *Eye, Ear, Throat, and Lungs.* Having for years studied anatomically, physiologically, and pathologically, all diseases of the human system, together with all malignant diseases of a tumefied character, such as Cancer, Fistula, and Piles, are *successfully* treated. DR. D. MAC RAE."

This though rather less bombastic and pretentious than most of those belonging to this class, yet affords an excellent type of the whole. The Doctor's simple office is dignified with the title of "Medical Institute," to create the impression throughout the country that he is at the head of some hospital, school, or other organized institution. Then comes the claim of having devoted "years of study," in some extraordinary way, to certain chronic classes of disease; and, finally, the positive assurance that all affections of that particular kind are "*successfully*" treated. To say that such advertisements are barefaced and deliberate frauds on the public, does not adequately characterize them. Like the one just quoted, they all include a pretence of special attention to, and ability to cure certain diseases that are in their nature, to a great extent at least, incurable; and are directly designed by their authors to attract the attention and delude by false hopes such persons as have been long afflicted and are ready to listen to any pretensions and make any pecuniary sacrifices that are accompanied by brazen assurances of relief. As an illustration, take the subject of cancer. A little boy is attacked with the most malignant form of the disease, in the bone of one side of his head and eye. Its location and nature show clearly to every intelligent and candid surgeon that it is totally incapable of remedy. The parents are truthfully informed, by their regular medical attendants, of the nature of the case, and of the palliative measures which are all that can benefit the patient. But just now comes under the eye of the afflicted parent a newspaper advertisement, in which the doctor claims cancer as among the diseases "successfully treated" by him. As a forlorn hope he is sent for. With pompous pretension, he sets at naught the opinions given by the intelligent and candid physician or surgeon who had attended the case, and authoritatively announces, that for fifty or one hundred dollars down, and as much more in a few weeks, he can remove the whole disease and have the patient restored to health. As drowning men catch at straws, so anxious parents, and adults weakened by disease, catch at every positive assurance, no matter how absurd. Consequently, the money is produced; the so-called doctor having been careful to secure the presence of a newspaper reporter, proceeds with the pretended operation, and the next morning we read, under the caption of "Important Surgical Operation," a glowing account of the removal of some formidable disease by Dr. M., after the patient had been given up by several distinguished surgeons. Thus the unprincipled charlatan pockets the poor patient's money, gets an editorial newspaper advertisement, in the form of a puff, and lies in wait for his next victim. Of course, in a few weeks, the duped patient finds his disease advancing more rapidly than ever to a fatal result, and silently sinks into his grave. But the newspaper report of the "Important Surgical Operation" is never corrected, and, consequently, its readers are left to wonder at the doctor's success, long after the poor patient is in his grave. It is surprising to notice the readiness with which newspaper reporters and proprietors are induced to become the medium of the grossest imposition upon the public, in relation to this class of cases. If they do it ignorantly, it is certainly high time they took measures to enlighten themselves. If they do it knowingly, for pay, then are they *particeps criminis*, in the meanest of all methods of obtaining money under false pretences. For it is difficult to conceive a meaner act than the deliberate deception of anxious parents or weakened and suffering invalids, for purposes of mere pecuniary gain.

The third class of advertisements, embracing those which the Society doubtless had more especially in view in appointing the undersigned committee, relate to the diseases and other consequences of licentiousness. It includes all those advertisements relating to "*Private Diseases*," the removal of "Female Obstructions and Weaknesses," and protection against too rapid an increase of families; whether they fill the columns of newspapers, or disgrace the fence posts, street corners, &c., or are thrown in profusion into public conveyances, or upon the doorsteps of public and private dwellings. They constitute a class of reading matter too disgusting to describe, and too demoralizing to be tolerated for a day in any well-regulated community.

There are three varieties of this class of advertisements, two of which are found in the columns of newspapers, and the other, in the form of handbills and pamphlets, is pasted along the streets, thrown into public conveyances, and left upon the doorsteps of private dwellings. They all possess, in a greater or less degree, three qualities, namely, false pretensions, obscenity, and either direct or indirect encouragement to vice and crime.

The false pretensions consist in, first, the most abominable lying in regard to the previous education, experience, and official positions of the pretended doctors, for curing private diseases; and, second, the positive and unqualified promise to cure permanently and speedily, "all the chronic and private diseases of the two sexes." Whoever opens a newspaper, and glances at these advertisements, and then remembers that not one of these advertising pretenders has any actual medical education, or has ever studied medicine proper in their lives, and that they are not recognized as belonging to the medical profession by any respectable physician in the country, will have some idea of their impudently fraudulent character. And yet who can estimate the number of victims, of both sexes, who are annually caught, and their pockets emptied by these very pretensions.

Their obscenity consists in the enumeration of every variety of disease arising from illicit sexual intercourse, and vicious sexual indulgences, with minute and vulgar descriptions of the sexual organs, sometimes illustrated by cuts. It is impossible to have these advertisements before the youth of both sexes, in the family newspapers, in handbills along the streets, in pamphlets and cards thrown into houses and yards, without making them more or less familiar with the most debasing vices and the most loathsome diseases; and that too, in such a way as to make the former appear only as "*youthful indiscretions*," or necessary appendages to human society, while the latter are stripped of all their terrors by the most positive assurances of speedy, safe, and permanent cures. And here comes in the strong encouragement given by these advertisements to vice and crime. The first step towards the indulgence in any vice or the commission of a crime, is to familiarize such vice or crime to the thought of the individual; and the second, is the positive assurance of immunity, or speedy relief, from whatever diseases or evil consequences may come from actual indulgence in the vice or commission of the crime. And in both these aspects, it would defy the ingenuity of man to devise anything more demoralizing to the community than the class of advertisements under discussion.

Their pompous pretensions and adroit enumeration and description of such diseases and organs as are calculated to awaken the curiosity of the young, are admirably calculated to attract the attention and familiarize the mind with thoughts of the most vicious character; while the deceptive assurances of sure and permanent cures, daily lessens the dread of consequences, until the vicious thoughts are recklessly accompanied by acts that leave upon the victim a moral and physical stain for life. But the most criminal quality of a certain variety of this class of advertisements remains to be noticed. We allude to those which directly and shamelessly encourage illicit and excessive sexual intercourse, both by the married and the unmarried, by promising safe and certain means for "*preventing an increase of family*," by either preventing conception, or procuring abortion. As a sample of this class, take the following, from the columns of one of our Chicago daily papers:—

"Highly Important to Married People.—New and Wonderful Discoveries in Physiology.—How to Control the Sex of Offspring, and *Prevent* an Increase of Family, with *Absolute Certainty*, without the possibility of failure, &c., &c."

No language can express the depth of depravity that dictates such advertisements, or the demoralizing effects they produce, especially upon the female sex. The fundamental idea set forth is, that free sexual intercourse may be indulged, while by the use of certain medicines, or the adoption of certain measures, the natural and ordinary consequences of such intercourse can be safely and certainly prevented. This idea, though utterly false in itself, has, nevertheless, gained such universal distribution and credence, during the last quarter of a century, that the number of painful and protracted uterine diseases, and the number of actual deaths, induced by the use of medicines and other means for either preventing conception or inducing abortion, is actually frightful to contemplate. Scarcely a week passes in any of our larger cities, that some female does not sacrifice her life as the direct consequence of such attempts, not one in twenty of which are ever known to the public, or outside of the immediate agents and attendants. The pernicious effects of the idea inculcated in these advertisements, and by a class of professional male and female murderers who, for $25.00 or $50.00, will undertake, deliberately, the work of fœtal murder, is not restricted to the production of a frightful amount of disease and death, but is plainly visible in the rapidly increasing repugnance manifested by married people, of both sexes and in all ranks of society, against bearing children or discharging the ordinary duties of parentage. It would require but a moderate amount of investigation, to show that this actually constitutes one of the most corrupting and destructive evils of modern society.

Having thus analyzed the character, and briefly stated the demoralizing and destructive consequences of what are usually styled *medical advertisements*, the next inquiry is, who are the parties responsible for their existence, and, of course, for their consequences also? The parties primarily responsible, are the authors of the advertisements, and the proprietors of newspapers who furnish the medium through which most of them reach the public. That newspaper publishers are morally and justly responsible for the character and influence of the advertisements they daily send to the firesides of their patrons, is as clear and certain as that the receiver of stolen goods is *particeps criminis* with the thief. They cannot plead ignorance of the character of the advertisements, for they are as much bound to know the character and influence of the advertising as of the editorial columns of the sheet they publish. The truth is, that Thompson, Bigelow, Whittier, Helmhold, or some other of the same genus, sits down and writes a quarter, half, or whole column of reading matter, in the form of an advertisement, containing the most glaring falsehoods, the most obscene allusions, and the most artful covering of loathsome vices, under the sugared phrases, "Unfortunate," and "Youthful Indiscretions," and, for a stipulated sum in money, the publisher deliberately puts the matter in print and sends it directly into from one to twenty thousand families daily or weekly, as the case may be. Now, by what rule of ethics, morals, or logic can the maker of the advertisement be held responsible for its legitimate effects, without including with him the publisher also? Advertising is, emphatically, the capital stock of the charlatan and nostrum vender. The proprietors and publishers of newspapers deliberately furnish this capital stock and share largely in the pecuniary profits. Can they escape an equal share of responsibility?

But while the makers and publishers are primarily responsible for their character and all the consequences that flow from them, in a secondary or more remote sense, our legislators, both State and Municipal, and our courts of justice are scarcely less so. For it is universally conceded to be the fundamental duty of the one, to enact such laws as will protect the lives and property of all classes of citizens, and of the other, to see that such laws are faithfully executed. And, certainly, in regard to no class of citizens, is this duty more sacredly binding, than in regard to such as are afflicted and enfeebled by disease. Practically, however, this is almost the only class, in our country, whose interests are left fully exposed to the prey of every species of pretension and fraud. If a man, by false statements, induces a merchant to sell him a bundle of goods on credit, he is amenable to prompt arrest and punishment. But if the unprincipled empiric sends, through the newspapers or by handbill, a totally false statement of his education, and ability to cure certain diseases, and thereby induces the invalid to employ and pay him for services that are worse than useless, he pockets his ill-gotten gains and continues the systematic work of deception with impunity. It is true, that we have upon the statute books of this State adequate laws for the punishment of fraud and deception, but it is evident that they were framed with direct reference to the transaction of ordinary business; and it is only by a judicial extension of the principles they involve, that they could be applied to the protection of the sick against the fraudulent pretensions of the advertising charlatan. It is the duty of our legislators to so revise the statutes on this subject, as to protect the sick with the same directness and efficiency as they do the merchant or the mechanic. We have, also, in the municipal laws of our city, a section prohibiting the exposure or sale of obscene, lewd, or demoralizing books, pamphlets, cards, &c., which fully recognizes the correct principle, that the community needs protection in this direction. But the language needs revision, to make the principle fully and practically apply to the suppression of all the advertisements and handbills relating to the diseases and consequences of licentiousness. That they are obscene in their nature, and, in their influence, corrupting to public morals, few reflecting men will deny. Therefore, laws for their suppression are as appropriate as laws for the suppression of licentiousness itself.

Hence, if, as we have endeavored to show, the whole series of medical advertisements relating to particular medicines, to doctors treating special diseases with extraordinary skill, and to the treatment of the diseases and other consequences of licentiousness are justly amenable either to the charge of attempts to obtain money by false pretenses, or to that of obscenity and corruption of public morals, and often to both, the remedy is obvious. It is, or should be, the duty of the prosecuting attorney of every judicial district, to present the authors and publishers of every such advertisement to the grand jury, for indictment and suppression. The recent case in the Recorder's Court, in this city, which resulted in imposing a fine of $100 on a notorious "private disease" doctor, for printing and distributing *obscene* pamphlets or handbills, shows that some, at least, of those occupying judicial positions, are ready to discharge their duty, in regard to the evils of which we complain. To bring about the proper legislative and judicial action, however, public sentiment, generally, must be awakened and corrected. For this purpose, it is the duty of members of our profession to more frequently expose, through the public press, such cases of gross imposition and fraud as are almost constantly coming under their direct observation. In furtherance of the same object, special efforts should be made to patronize and sustain the small number of newspapers and periodicals that actually exclude from their columns all such advertisements as are alluded to in this report. This would strongly encourage an increase in the number of such papers, until newspaper proprietors, generally, would ultimately find their *pecuniary* interests coinciding with their moral obligations in relation to this subject.

N. S. DAVIS,
*Chairman.*

